# Wonder and the Patient

**DOI:** 10.1007/s10912-014-9320-6

**Published:** 2014-12-09

**Authors:** H. M. Evans

**Affiliations:** 1 Centre for Medical Humanities, Durham University, Durham, UK; 2 Trevelyan College, Durham University, Elvet Hill Road, Durham, DH1 3LN UK

**Keywords:** Wonder, Patient, Well-being, Embodied agency, Medical humanities, Health geography, Imagination

## Abstract

Is it possible to distinguish, as sociologist Arthur Frank proposes, an ‘ideal of wonder’ within which ill persons could recover some of their former sense of life and flourishing, even within the constraints of ill-health? Beyond this, are there more general benefits in terms of health and well-being that could accrue from cultivating an openness to wonder? In this paper I will first outline and defend a notion of wonder that gives philosophical support to Frank’s proposal, noting why thinking about medical treatment may readily provoke a sense of wonder. Second I will however limit the normative force of such an ‘ideal of wonder’ noting its demands and some of the challenges facing it. The paper goes on, third, to conjecture wider benefits within and beyond the clinical encounter arising from being mindful of the wonder of embodied human agency. Fourth the paper will consider alignments between the foregoing analysis and some theoretical commitments in recent work in health geography. Finally I will briefly reconsider the notion of the body-as-territory, and the role of the imagination in bringing it under wonder’s gaze.

## Introduction - Arthur Frank’s ‘ideal of wonder’

At the age of forty the sociologist Arthur Frank developed seminoma, a form of cancer. During the course of his illness he encountered and developed a strong sense of wonder at his own body, and learned to take from this sense of wonder both solace and inspiration (Frank 2002, 50–63). He urged such a sense upon other patients and also upon his own and other medical carers. Elsewhere I have given consideration to the relevance of a sense of wonder to clinicians (Evans 2012); here I would like to turn my attention to the patient. Frank’s story invites just such attention, not because he gives a particularly thorough or analytic account of what wonder is — he doesn’t — but because wonder arises for him as a compelling experience in the circumstances of serious illness. Those are circumstances of which he is able to write with great authority both as a distinguished scholar and as an acute witness in his own case.

I want to begin by drawing on his own reflections upon the salient part of the story. Describing his experiences of becoming progressively ill, submitting to tests, entering hospital, undergoing surgery, and beginning to recuperate, he comes face to face with the challenge of having as it were to negotiate both with his ill body and with his professional carers. Medicine’s instincts were to seek to control his illness *through controlling his body*:

Every day society sends us messages that the body can and ought to be controlled. Advertisements for prescription and non-prescription drugs, grooming and beauty advice, diet books, and fitness promotion literature all presuppose an ideal of control of the body. Control is good manners as well as a moral duty; to lose control is to fail socially and morally. But then along comes illness, and the body goes out of control. … Physicians justifiably think it is their duty to restore, in the name of society, the control that the sick are believed to have lost. Control, or at least management, becomes a medical ideal (Frank 2002, 58).

Frank’s experiences of medicine’s attempts at control were discouraging. Partly this arose from correctable deficiencies in procedure (and in common courtesy) in the institutionalised healthcare he received; but partly it arose from the fearful realisation that medicine might dissemble over something that he would ultimately have to acknowledge openly, namely that cure might not be possible. The resources he needed had to include an attitude towards his own ill body that was grounded on something other than its subjugation. This attitude, it transpired, was one of wonder at the body: a wonder held on his part certainly, but also, ideally, on the part of his medical carers:

What I recommend, to both medical staff and ill persons, is to recognize the wonder of the body rather than try to control it. Wondering at the body means trusting it and acknowledging its control. I do not mean we should stop trying to change the direction the body is taking. I certainly did all I could, and I value all that my physicians did, to use treatment to change the direction my body was taking. Wonder and treatment can be complementary; *wonder is an attitude in which the treatment can best proceed.* (59, my emphasis)

Illness might simply be something he had to live with, but an attitude of wonder could make this meaningful.

Wonder is almost always possible; control may not be. If the ill person can focus on an ideal of wonder in place of control, then *living in a diseased body can recover some of its joy.* (Frank 59, my emphasis)

Frank’s experience is striking. But not only that: as he describes it, it is also heartening (irrespective of the fact that, fortunately, he also enjoyed a good clinical outcome: seminoma is one of the more treatable cancers). However, is his advocacy of a sense of wonder realistic? To what benefits or goods might it credibly give rise? (In particular, how does it help treatment to ‘best proceed’?) And how widely might one really urge this ‘ideal of wonder’ upon other patients? In proposing it, Frank is obviously making a normative claim, but he doesn’t indicate its strength; in particular he doesn’t tell us whether this is something that we can ordinarily *urge upon* patients, as distinct from applauding it when we happen to find it in them. Nor does he spell out what he takes wonder and wondering to consist in, as exercised in the circumstances he envisages. I think these are solid questions worth pursuing, and it is in an appreciative spirit that I will try to do so.

First, I will briefly outline a notion of wonder that I believe is substantial, supports Frank’s proposal (albeit from a philosophical rather than a social-scientific perspective), and is relevant more widely to the clinical encounter — including those in general practice as distinct from hospital medicine. I will also note why thinking about medical treatment readily provokes wonder. Second, as for the normative force of the ‘ideal of wonder,’ I will be prepared to limit it. It is an ideal that is initially difficult to approach, and relatively few patients may habitually feel wonderment in relation to their own embodiment, ill or otherwise. This doesn’t make Frank’s ideal any the less worthwhile: the occupants of minor roles deserve, and may well reward, study. So, third, I will conjecture what further good things might follow (mainly, but not exclusively, for the clinical encounter) when a patient is indeed mindful of the wonder of embodied human agency in general and of their own in particular. (Frank’s is fundamentally a patient-centred view; my own analysis is situated between the perspectives of patient and physician.) Fourth I will consider the extent to which the analysis offered here aligns with some of the theoretical commitments underlying some recent work in health geography. Finally I will briefly reconsider the notion of the body-as-territory, and the role of the imagination in bringing it under wonder’s gaze.

## Wonder and treatment

Wonder, for Frank, seems to be a specially-reassuring mindfulness and attentiveness to one’s circumstances, both mediated through and often focused upon the body — ill or not. It is clearly and importantly positive: it is a source of recovered joy for the ill person, a manifestation of trust towards to the body, and a vitally enabling circumstance, ‘an attitude in which treatment can best proceed’(59).

I certainly endorse its being an attitude rather than an emotion (Evans 2012, 85) — one of intensified and compelling attention, in which the ordinary is presented to us anew, commingled with and suffused by aspects of the extraordinary; our imagination is engaged in advance of our understanding though that might follow (Evans 2002, 127). Wonder is, I would argue, more profound and more durable than curiosity; for instance, it survives explanation: the wonder of the universe is frequently increased, not diminished, with our understanding of its inter-penetrated complexity. Its objects need not threaten to impact upon us as might those of awe; shorn of an aesthetic, it need not engage the sublime or the terrifying (Evans 2012, 127). It can be both impersonal (especially not directed at the self [Moore 2005, 269]) and intimate — think of the simple ‘tangled bank’ upon which Darwin saw written the entire story of evolution (1859). Wonder is above all a glimpsing; a disclosure (Miller 1992, 51).

This view is, I believe, echoed in Frank’s description of its source on the occasion that concerns him (and he mentions no precursors): it seems to have come about through his defiance of the physical misery of his illness by enjoying a uniquely vivid experience of walking in the September rain, which on the occasion in question he valued for its own sake (not least, perhaps, because of the possibility of its impending loss); crucially, he was undistracted by any plans, purposes or inward-facing concerns. Perhaps through the mediation of intense experiences, wonder for Frank derives from the body, from which it was ‘learned’ (59) and for which he notes that he can claim ‘little credit’ (59). The embodied ground of experience of all kinds, wonder included, seems inescapable; it is understandable that his focus is upon the ill body that he has become. I tend to think that wonder swiftly proceeds outside ourselves, taking us away from ourselves towards ‘something in the face of which we set aside our own concerns and even our self-conscious awareness, in the most powerful instances’ (Evans 2012, 127). In wonder, we find the world, and ourselves within it, temporarily ‘transfigured’ (there is a measure of consistency among the views emanating from the relatively narrow range of disciplines that have considered wonder from a scholarly viewpoint [Evans 2012]). That ‘green September day’ was, I suspect, *transfigured* for a while.

Now consider the provocation of wonder in relation to treatment: if transfiguring is essentially a matter of perception, treatment aims at substantial material change: ‘It is no small thing to have your body rearranged, first by disease and then by surgical and chemical interventions intended to cure that disease’ (54).

When we can pause and acknowledge it, wonder is dramatically invited by our bodies; by their constitutions, complexities and capacities; and by their being the material fundament and medium of that extraordinarily elusive yet ever-present song of life, daily conscious experience. Few things spotlight this more dramatically than medical treatment (Evans 2014).

Consider, first, what it is to be doing nothing in particular, thinking and feeling nothing in particular, in no discomfort or agitation but no especial pleasure or stimulation either — simply in the ‘background’ state of being ready to do or think the next thing that comes along needing to be done or thought, the unnoticed small change of bodily existence ready to be cashed out in judgment or action. This is the taken-for-grantedness of ordinary being, and it could last not a moment without the extraordinary complexity and frantic silent activity of the bio-physico-chemical constellation that is our bodies. ‘Health is life lived in the silence of the organs,’ says Leriche (quoted Canguilhem 1989, 91). Woven into and emergent from this constellation, its patterns as reliable as they are intricate, are our ordinary perceptual and kinaesthetic and proprioceptual experience; our qualitative sensations; our capacity for recognition and memory, movement and conjecture, decision and willed action; in short, our embodied agency. If we are aware of any of this at all, ordinarily it is only subliminally as we foreground only what is of concern to us. ^1^

Consider, second, that upon this improbable fabric are wrought extraordinary metamorphoses: of an ordinary life-cycle; of disease and recovery; and — this the intelligent *purposive* work of other embodied agencies — of the treatments that are medicine’s names for its own organised changes in our constituent flesh. These bodily ‘rearrangements’ are indeed ‘no small thing,’ in either experiential or, when we stop to think keenly about it, metaphysical terms (Evans 2014). Successful or not, medical treatments provoke wonder and they constitute wonders — albeit usually unattended-to. (Indeed, illnesses might also provoke wonder for some patients.) ^2^

I have elsewhere suggested how this provocation can be important in the clinical encounter especially from the clinician’s viewpoint (Evans 2012). Wonder conceived as a transfiguring attentiveness offers to the clinician an ever-present source of ethical regard (since even the most damaged patient retains embodiment, always worthy of wonder, in a context that plausibly joins wonder to respect); a sense of wonder can revitalise diagnostic imagination amid the dulling impact of clinical routine; and through wonder at embodied human experience, the clinician may find irresistible the recalling of her shared vulnerability with the patient. It is to both the first and the last of these, to ethical source and to shared vulnerability, that I suspect Frank implicitly appeals when he says that ‘The body is not a territory to be controlled by either the physician’s treatment or the patient’s will’ (Frank 2002, 62).

The inference we may draw is, I think, that for Frank wonder stands in an ambiguous relationship to treatment. Wonder invites us to attenuate our dependence upon treatment when this is too-readily misconceived under the ideal of control. However, Frank endorses treatment in his commending wonder as that attitude in which treatment may ‘best proceed.’ Perhaps wonder is ambiguous like this: perhaps both aspects are true.

## The normative force of the ‘ideal of wonder’

The very term ‘ideal’ is almost paradigmatically normative — that at which one should most perfectly aim — but it is a term often used in intentional contrast with the practical world, and different ideals (like different norms) often compete: indeed, the ideal of wonder is urged by Frank precisely as a rejection of the ideal of control. He is under no doubt about the significance of wonder to the clinician:

A physician who does not have this sense of wonder seeks only to cure disease. Sometimes he succeeds, but if cure is the only objective, not achieving it means he has failed. For the artful physician, wonder precludes failure. The physician and the ill person enter into a relationship of joint wonder at the body, in which failure is as irrelevant as control. (62)

It seems hyperbolic to suppose that ‘wonder precludes failure,’ except in the rather stipulative sense of suppressing cure as a goal whenever cure seems beyond us. A sense of wonder might indeed give us a supplementary reason for doing so, perhaps through giving us a way of seeing the patient’s world anew (and seeing anew is a characteristic of a state of wonder), but wonder has no monopoly upon our recognising that other goals sometimes supplant the aim of cure.

Frank’s conjecture that wonder is ‘almost always possible’ (59) seems equally ambitious; it is a surprisingly large claim in those terms, and perhaps we should infer it to mean rather that wonder is almost always possible for those who already know how to access it. For a sense of wonder is something that someone may or may not have in ready response to a particular situation: Frank is openly troubled by the difference between those doctors who in the clinical context have it and those doctors who do not, insofar as this determines whether they might share a sense of wonder with the patient.

As it stands, Frank’s view of wonder as both importantly beneficial and readily available is strongly normative. Yet, gripped as I am myself by the call of a sense of wonder in response to human embodiment, I am not at all certain how generally I could expect everyone to share it. This is a matter quite separate from how good I think it would be if they did (generally I would think it a very good thing indeed). Rather as risks are properly understood as a combination of two independent variables, the magnitude and the likelihood of the harm in question, so perhaps normative force might — at least, here — be thought a combination of both the scale and the attainability of the good in question. To the nature of that good we shall shortly turn, but I think its attainability is subject to a limitation that in turn limits the normative claim of wonder as an ideal. People vary in their inclination towards wonder according to habit, disposition, even talent (Opdal 2001) not to mention the filtering of sensitivity and imagination by upbringing (education and developmental psychology are alike foci of disciplinary enthusiasm for studying wonder [Minney and Potter 1984]). Indeed, were modern industrial societies and their institutionalised healthcare systems not more orientated towards the ideal of control than that of wonder, Frank would not be saying anything distinctive and nor would he have felt the need to say it. ^3^ Thus, habitual openness to wonder may be no more easily producible from a standing start than is, say, humility or compassion; this does not undermine them as normative ideas, but it might temper our expectations.

This importantly distinguishes clinicians from patients — and recall that Frank commends wonder to both groups. I strongly believe that for physicians an openness to wonder is an educational good that could well be promoted in aspects of the medical curriculum. However, while wondering (and empathetic, and humble, and compassionate) clinicians can be encouraged in these respects, they are more plausibly identified and encouraged by medical schools’ admissions processes than they are manufactured by their courses of instruction. But no such selection process applies to patients whose vocation is first and foremost an inevitability of the frailty of flesh. Frank is, in my view, right to commend wonder to both physicians and ‘ill persons,’ but it might be only a minority of patients — well or ill — who are naturally disposed to respond to his call, at least at first. Frank’s own experience notwithstanding, serious illness may for many patients be not at all the right time to start to develop a sense of wonder: for many, the very *illness* of illness may preclude it.

None of this makes Frank’s claim any less important, nor the ‘goods’ made available in wonder any less valuable, but it does remind us that the claim’s normative force is limited. With this acknowledgement, let us now consider what good things might indeed follow, where a patient is open to wonder.

## Some ‘goods’ arising from a patient’s sense of wonder

### Recognising shared embodied agency

I recalled above that the physician who is open to wonder at embodied human nature is thereby also mindful that she shares this nature, and its attendant vulnerability, with the patient. The patient who is open to wonder can reciprocate that sense of shared nature and vulnerability. Patient and physician may thus jointly recognise the intensity of the physician’s task, and the commitment (to helping the patient who may be the locus of significant suffering) and understanding (of that same shared fragility of the flesh) that this task requires of her.

I suspect Frank has exactly such reciprocity in mind when he observes that ‘The ill person who finds a physician to join in this wonder is fortunate’ (62). This might mean that such fortune is rare as well as good, of course. Both the rarity and the goodness are acknowledged in Michelle Clifton-Soderstrom’s attempt to ground the ethical foundation of medicine in the reciprocal ‘otherness’ of other people. In this account, interpersonal relationships (including, specifically, the clinical relationship between patient and physician) have a primary ethical and ontological character that precedes the ‘knowing’ relationships of science: ‘the first encounter with the Other is not one of comprehension’ but one of ethical recognition and acknowledgement (2003, 450). Signally, Clifton-Soderstrom grounds this squarely in *wonder*, implicitly of a self-diminishing kind that she contrasts with something more akin to scientific curiosity. As she puts it, the otherness of another person transcends the idea of ‘the Other’ in oneself,

…point [ing] to the phenomenon of *wonder of another person*, and wonder as the experience that distinguishes human beings. The experience of wonder is often neglected in the practice of medicine. Instead of a profession filled with the wonder of who the Other is, scientific wonder becomes the main or only parameter for medical practice. (453, my emphasis)

It may be that both physician and patient sense the *existential*, rather than simply scientific, wonder of embodiment and the wonder of addressing it. But even if that recognition be a vibrant one only for the patient, the clinical encounter can still in consequence be a more mutually-respectful occasion, surely a good thing for patient and clinician alike, and it becomes an occasion with greater imaginative possibility — increasingly important with the rising proportion of presenting cases (particularly in family practice) whose problems are ‘functional disorders’ and whose basis, being at least in part emotional or social, may require correspondingly more imaginative investigation (Muller-Lissner et al. 2001). If we are looking for merely instrumental goods from the patient’s having a sense of wonder, then our first operative example seems to lie within the clinical encounter.

### Shared commitment to the clinical endeavour

A second gain, more obviously featuring in the clinical context, will be a consequence of the first. An appreciation of and respect for his own (and others’) bodily powers and limitations combined with an intensified appreciation for the clinician’s engagement with those limitations in his own case seems likely to result in the patient’s having a *more strongly shared commitment to the clinical endeavour*. (Whether or not the greater imaginative freedom, that one might think implied by an openness to wonder, tends towards the giving of a richer history rather than a merely more unfathomable one is, of course, a separate question.)

This notion of shared commitment is obvious enough and easily understood. However, the evident reciprocity that it involves on the patient’s part seems too easily eclipsed by the predominant focus upon the physician’s commitment and obligations, and I think this asymmetry is worth a little attention. Clifton-Soderstrom, for instance, draws her conclusion in terms that rest mainly upon the physician’s need to recognise the otherness of the patient as constituting the prior grounding of ethical response. Her analysis is an application to clinical medicine of the ethics of Emmanuel Levinas (2003, 451). But it seems to me that this cuts both ways in the clinical encounter: to the *patient* the Other is the physician whose own otherness must alike be respected. This requirement may understandably be dimmed in the context of a patient’s significant suffering. However in ordinary workaday clinical consultations — perhaps most especially in primary care — I see no reason suddenly to drop this reciprocal requirement of acknowledgment of the Other simply because the project of the clinical encounter is an asymmetric one (that is, primarily conceived towards the benefit of the patient). Even the context of the clinical encounter *as* clinical is, on the story that Clifton-Soderstrom is presenting to us, epistemically subsequent to the primary ethical context of an encounter between two people who are *reciprocally* Other. All the more reason then to emphasise that the patient open to wonder at his own embodiment is likely to feel more fully a part of that endeavour and to behave as a more engaged partner in discerning and fostering the means to his own recovery.

When, as sometimes happens, the patient’s conception of his needs differs sharply from the physician’s, then a demonstrably shared sense of responsibility can only improve the patient’s chances of persuading the clinician to take seriously his dissenting view and to respect it.

These first two goods are ‘instrumental’ in that they subserve other goods or goals. But it is possible that there may be something intrinsically, constitutively, good about having a sense of wonder. An ‘ideal of wonder,’ like an ‘ideal of service,’ suggests something whose virtue cannot simply be reduced to the accumulated virtues of its good results.

### Wonder as constitutive of a flourishing life?

One way of responding would be to think in terms of what constitutes a flourishing life: what goes into a life — any life — that in some recognisable sense ‘goes well’? There is no need here for any essentialist attempt to specify necessary and sufficient conditions for a flourishing life. We need simply identify things whose inclusion in a life would typically make that a better life than it would have been without them. An otherwise sustainable life that additionally has such things is, to that extent, also a flourishing life. Examples might include: kinship; friendship; a sense of the community to which one belongs; a sense of one’s identity and purpose; laughter; imagination; intimacy; beauty; the ability to make enduring meaning; and doubtless many others besides. I claim that we can readily add ‘a sense of wonder’ to the list, where — in line with the account we gave above — this sense means the inclination and ability to remain imaginatively open to the fullness of the world around one beyond the reach of immediate explanation. It is reasonable to prefer a life in which wonder is possible to an otherwise corresponding life in which it was not: this is not because the ability or inclination to wonder *does* or *makes* or *supports* anything else in particular, but simply because — like kinship and imagination — it is *prima facie* a good thing to have, engage, enjoy. Thus wonder does not simply support a flourishing life: rather, it part-constitutes one; it is not so much a route to flourishing: rather it is part of what it is to flourish, one flavour of what Nussbaum would call a ‘sense of life’ (1987). ^4^ Additional work is needed, both in making a general case and in quarantining exceptions, but I would expect such work to yield a strong argument in favour of including a sense of wonder — or, in Frank’s terms, an ‘ideal of wonder’ — among life’s intrinsically good things. Indeed, it invites a variation on Socrates’ famous dictum concerning the unexamined life: “The un-wondering life is less worth living than one lived in wonder.”

### Wonder as constitutive of a healthy life?

Is openness to a sense of wonder more specifically constitutive of a *healthy* life? This is more elusive. It would look more plausible as we construed ‘health’ more broadly — in effect, the more that we took health to converge upon flourishing. But the objections to such convergence are substantial and well-known. A healthy life cannot be *eo ipso* a flourishing one, since not all flourish who enjoy good physical health or vice versa; and a more fundamental problem concerns the limitless authority and responsibility for flourishing that such convergence then appears to place upon medicine. In any case, Frank’s own narrative distinguishes clearly medical and also clearly non-medical dimensions to his illness and recovery; this is not a route that could support his account.

A healthy life sounds like (and indeed presumably is) an intrinsically good thing, other things being equal; so now we need to know how remaining open to wonder counts as an integral part of the healthiness of that healthy life. But to maintain that a sense of wonder is itself a ‘healthy’ thing is either to present it as *conducive* to health in the particular ways that we have already considered or more adventurously to align it with other clearly existential goods such as kinship, intimacy, fulfilment and so forth that are often regarded as health-sustaining or health-promoting (and then to seek out the supporting evidence [White 2009]) or to speak metaphorically. Frank’s account suggests that he might endorse the first and the second of these; whereas relying on the third usually means that all analytic bets are off.

## Alignments and misalignments: geographers and wondering

In some respects my discussion may appear to echo, even to align with, aspects of the ‘turn to affect’ that engages much thinking in geographical writing, including health geography writing. ^5^ The key aspect here is the centrality of embodiment in experience. I have noted that wonder is both mediated through and focused upon the body, even (for Frank) ‘learned from’ the body. I have conjectured *inter alia*: that in wonder our imagination is engaged in advance of our understanding; that (*pace* Clifton-Soderstrom) existential considerations are pre-eminent over scientific ones in addressing the wonder of embodiment; that ordinary being is taken-for-granted rather than analysed; that our bodies are the medium of daily conscious experience; and that willed action is not wholly distinguishable from other aspects of embodied agency — agency which itself, taken as a whole, is often only subliminally available to our conscious awareness, and is in some respects forever mysterious. In the same spirit, I contrast imaginative awareness with immediate explanation.

This much appears in some respects to align with some of the tenets of ‘non-representational theories’ in geography. In particular the emphasis on situating our view of understanding (epistemology) in the context of our material nature (ontology) might seem to sit well with non-representational theories’ concern to enfold the (otherwise detached, external) objects of experience and symbolic representation into an essentially acted, lived, bodied world of practices and inhabitings. Ben Anderson and Paul Harrison summarise this concern neatly thus: non-representational theories ‘share an approach to meaning and value as “thought-in-action”’ (2010). States of wonder might be taken to be particularly intense illustrations of what Anderson and Harrison dub ‘constant relations of modification and reciprocity with their environs’ (2010, 7). Or again NRT’s emphasis on *relational* ontologies (Bissell 2010) might provisionally seem echoed in my grounding the ethical foundation of medicine in a mutual otherness and in my suggestion of the self-abnegation of some intense states of wonder. And Greenhough’s insistence on ‘understanding the world through an engagement with its materiality’ (2010, 43) appears *prima facie* to offer the very stuff of some of the most intense wondering experiences (though unfortunately experiences that are not considered in this paper), concerning the very ipseity, the very ‘this-ness’ of things that reminds us of what Wittgenstein regarded as the most fundamental of philosophical mysteries, that there is a world at all, that there is anything at all rather than nothing (1961, section 6.44).

But there are crucial misalignments, too. Within Frank’s reported experience, and lurking too in my analysis, lies an aspect of stubborn residual dualism — the self that *he* is, wondering at *his* body, temporarily externalised or detached — and I do not see that this can (or should) be fully dissipated. Moreover, wonder more generally seems in one sense highly externalising. One’s reaction to the thing wondered-at may be deeply embodied as well as deeply imaginative, but at the very least, wonder is liable to concern the world-out-there as something that is really present, in order that it may also be, briefly, newly-present. This declares wonder’s intentionality, which seems to me to be of its essence: in wonder, one wonders-*at*; one does not simply ‘wonder’ *tout court.* I have no grasp of states of wonder that are void of intentionality and no sense that such states are even coherent.

More generally, my analysis neither requires nor can tolerate very much subordinating of meaning or significance. Anderson and Harrison argue for a sophisticatedly altered priority among the sources of meaning rather than for meaning’s wholesale relegation, but some of their predecessors in theory may be less cautious. Thus the criticisms directed at Nigel Thrift among others, in Ruth Leys’ sustained scepticism concerning the ‘turn to affect’ and the dangers of ontological privileging of ‘corporeal affective reactions’ over cognition and intentionality, which are reduced seemingly to little more than epiphenomena (Leys 2011). *Pace* Leys, the avowedly embodied grounding of experience does not seem to me even momentarily to entail that its intentionality must, or even could, be dispensed with. More specifically, I believe that wonder is conceived and encountered in response to (or even as a form of) the representation of the thing wondered-at; the world is made ‘newly-present’ in changed representation and signification. What less hard-line affective theorists generally, or non-representational theorists in particular, might make of wonder *other than* as an intense, atypical, form of embodied attunement is not immediately clear, but the question could supply an intriguing dialogue to be pursued with them.

Elsewhere, perhaps contemporary geographers more readily engage with considerations of curiosity than of wonder; while they are related notions, curiosity has a more obviously teleological nature than does wonder, necessarily *constituting* rather than contingently provoking a desire to understand. Ironically, even curiosity has been too often dismissed as insufficiently practical in, for instance, discourses about academic impact, something that Richard Phillips laments in the course of advocating resistance through ‘geographies of curiosity’ in which for instance geographers can ‘illuminat[e] the ways in which environments variously encourage and discourage curiosity’ (2010, 448) and by dispelling anxieties ‘about emotional dimensions of academic practice’ (449). Neither curiosity nor wonder is, to my mind, plausibly an emotion, but securing physical, intellectual and moral spaces that are conducive to curiosity may invite further analysis about whether there can be geographical underpinnings to attitudes of wonder. If so, such attention might valuably be paid to the clinical, ancillary and social spaces of healthcare, and to those characteristics of spaces (clinical or otherwise) that might be able to encourage untrammelled, rather than trammelled, reflective experience. Phillips also makes a valuable point about the plainly geographic savour of descriptions of curiosity that are couched as exploring unknown territories, though whether states of wonder are so readily captured in geographically-orientated terms needs further thought (449). More generally, geographical engagement with wonder may be a focus for interesting future work although the most obvious geographical *sources* of wonder of the kind celebrated by Keats — the prospect of Cortez staring with ‘eagle eyes’ upon the Pacific, ‘Silent, upon a peak in Darien’ — sadly belong to a world less-charted than our own (Keats 1884).

## Wonder, the body and imagination

As we’ve noted, Frank declares that ‘The body is not a territory to be controlled by either the physician’s treatment or the patient’s will’ (62) in justification of the hope that for ‘the ill person [who] can focus on an ideal of wonder in place of control, then living in a diseased body can recover some of its joy’ (59). Frank’s view combines an overt ethical programme with an ontology that is only implicit and ambiguous; objectors to dualist accounts of our embodied state will find only correspondingly ambiguous support here, and there are echoes of the notion of our ‘inhabiting’ our bodies that cannot be entirely stilled.

Yet it seems to me that our experiences of embodiment *are* ambiguous in this way. Our identities are grounded in such continuities as we (and those around us) can most readily find. Dementia and other erosions of our cognitive and interpersonal faculties can amount to wholesale transformation within an apparently unperturbed embodiment. However, diseases such as the cancer that Frank faced can bring about physical metamorphosis of the gravest kind — accelerating or distorting aspects of the metamorphosis of our typical embodied lifetime careers. Disease and medicine’s response do, alike, achieve changes in us, but they also achieve changes in what I’ve called ‘our constituent flesh;’ in the throes of the struggles involved, the hoped for ‘changes in the direction that my body was taking,’ as Frank put it, we do indeed inhabit our diseased bodies even while we hope for their restoration.

At the very least this is the dualism of relation: we may succeed in replacing the ideal of control with the ideal of wondering-at, but both are attitudes towards the body: both *address* the body from a position that is somehow both immanent within, and transcendent towards, the body in question. For me this is a further ground of wonder — metaphysical wonder - but I notice in more prosaic terms that it relies heavily upon our acts of imagination.

Imagination is very much a mode of agency. Frank’s commending the ideal of wonder consists throughout in emphasising agency — he commends and urges a sense of wonder as itself active and as something that we can attain through learning and development. It was an act of the imagination that disclosed to him what, he feared, medicine was implicitly hiding — that cure might not be possible. It is a forward-looking imagination that conjectures wonder as an attitude ‘in which treatment can best proceed’ — something for whose first experiment in any individual’s case there can hardly be prior evidence. Reading about Frank’s experience could prepare me for facing a comparable adversity; but when the time came to put his thesis to the test, I would have nothing more solid than an act of faith and imagination. And indeed any reasonable analysis of wonder as an *ideal* — something to be elevated, generalised, striven for even if not actually attained — requires our imaginative projection. The contrast between the attunement involved and the workaday entrapments of lazy or fearful thinking constrain the imagination as they constrain the sense of wonder more generally.

This does not make a sense or an ideal of wonder at the body any less important — simply more demanding of our imaginative energy. I can think of no more worthwhile expenditure of that energy than in pursuit of Frank’s implicit view that a sense of wonder, like a sense of beauty, is a transfiguring ideal capable of presenting the world to us anew, in health and in sickness alike.
